# Intraosseous administration into the skull: Potential blood–brain barrier bypassing route for brain drug delivery

**DOI:** 10.1002/btm2.10424

**Published:** 2022-10-21

**Authors:** Ji Hee Kang, Young Tag Ko

**Affiliations:** ^1^ College of Pharmacy, Gachon Institute of Pharmaceutical Sciences Gachon University Incheon Republic of Korea

**Keywords:** BBB‐bypassing route, brain drug delivery, brain uptake, CNS disorders, intraclavariosseous

## Abstract

Progress in treating central nervous system (CNS) disorders is retarded owing to a limited understanding of brain disease pathology. Additionally, the blood–brain barrier (BBB) limits molecular entry into the brain. Many approaches for brain drug delivery to overcome the BBB, such as BBB permeability enhancement, transient BBB disruption, and direct surgical administration have been explored with limited success. Recent research has shown that direct vascular channels exist between the skull bone marrow and the meninges, allowing myeloid and lymphoid cells to migrate. We hypothesized that these direct channels may also allow brain drug delivery from the skull bone marrow to the brain. In this study, for the first time we propose intraosseous administration of drugs into the skull (intracalvariosseous [ICO]) as a novel approach for brain drug delivery via BBB bypassing routes. We tested the feasibility of the approach by applying nine representative compounds over thinned mouse skulls to simulate ICO and measuring the compound entry level in the brain compared to that after systemic administration. Surprisingly, we found that the skull is not completely impermeable to drug penetration into the brain and the tested compounds reached the brain tissue several tens‐to‐hundred times higher by ICO than systemic application. These findings suggest a role for the BBB bypassing route from skull to brain, apart from the systemic route, in the drug entry into the brain after ICO. This approach should be applicable to other CNS drugs and even BBB impermeable drugs. Overall ICO provides an innovative and advantageous pathway for effective treatment of brain diseases.

## 1 INTRODUCTION

Central nervous system (CNS) disorders constitute approximately 6.3% of the global burden of disease.^1^ Despite the progress made in the last few decades in understanding the brain, including the structure and functions of CNS, novel drug discovery and its clinical development in treating CNS disorders are still major problems in the neuro‐pharmacological sector.^2^ The probability of in‐development CNS disorder drugs being commercially available is approximately two times lower, whereas the development and approval period is approximately two times higher, than that for other disease drugs.^3^ The reasons for the low therapeutic success of CNS drug development include minimal or no understanding of the relevant pathophysiology of CNS disorders, difficulties in developing preclinical models and assessing target engagement, the presence of the blood–brain barrier (BBB), CNS‐mediated side effects, the lack of clinical scales of sensitivity, and interference with the therapeutic benefit of placebo effects.^4^, ^5^, ^6^

The major obstacle to drug entry into the brain is the presence of the BBB, composed of endothelial cells linking tight junctions.^7^ The BBB, with its distinctive structure, maintains homeostasis by regulating efflux and influx, and protects the brain from pathogenic agents.^8^ To overcome the BBB, approaches such as enhancement of BBB permeability, transient BBB disruption, and local direct administration by surgery have long been on the list of possible clinical practices.^9^, ^10^ Various strategies involving the design and modification of active drug molecules and nanomaterial‐based drug delivery have long been studied to enhance BBB permeability. In addition, the transient opening of tight junctions using hyperosmotic solutions, focused ultrasound, and electromagnetic fields can potentially permit the entry of CNS drugs into the brain in efficacious amounts.^10^ Although significant progress has been made in overcoming the BBB to deliver CNS drugs to the brain, many approaches have provided limited clinical success.

Recent findings demonstrate the existence of direct ossified vascular channels between the skull bone marrow and meninges, allowing myeloid and lymphoid cells to migrate.^11^, ^12^, ^13^, ^14^, ^15^ Research has shown the presence of hundreds of capillaries known as trans‐cortical vessels (TCVs), which form a direct connection between endosteal and periosteal circulation. Furthermore, structures similar to TCVs in flat calvaria bone were identified, thus confirming the presence of direct channels between the bone marrow and brain surface.^11^, ^16^ Additionally, it was demonstrated that cerebrospinal fluid (CSF) enters skull bone marrow niches in physiology and pathology, where it modulates myelopoiesis and egress to meninges.^17^ We speculated that these channels could be bi‐directional, enabling bone marrow to have direct access to the CSF. Furthermore, we hypothesized that these direct channels may also allow drug delivery from the skull bone marrow to the brain.

In this study, we propose for the first time, the intraosseous administration of CNS drugs into the skull (intracalvariosseous [ICO]) as a novel approach for brain drug delivery via BBB‐bypassing routes. Our goal was to verify the clinical feasibility and evaluate the comparative advantage of ICO over systemic administration using quantitative pharmacokinetic analysis. We expected that the highly porous diploes and permeable cortex of the skull bone^18^ would allow CNS drugs applied into the diploes to cross the cortex to the brain parenchyma. Brain drug delivery through the ICO administration route is completely different from various attempts at overcoming the BBB.

## 2 MATERIALS AND METHODS

### 2.1 Materials

Chlorpromazine hydrochloride (CPZ), risperidone (RIS), temozolomide (TMZ), paclitaxel (PTX), donepezil (DPZ), and rivastigmine l‐tartrate (RVG) were purchased from TCI (Tokyo, Japan). Risperidone‐d4, γ‐aminobutyric acid‐d6 (GABA), and glutathione (glycine‐13C2, 15 N) sodium salt (GSH) were purchased from Toronto Research Chemicals (North York, NY, USA). [UL‐^13^C_12_]sucrose (all 12 carbons labeled with ^13^C, SUC), [UL‐^13^C_6_]sucrose (only six carbons in the fructose moiety labeled with ^13^C), D‐[UL‐^13^C_6_]glucose (all six carbons labeled with ^13^C), and D‐[UL‐^13^C_6_]fructose (all six carbons labeled with ^13^C) were purchased from Omicron Biochemicals (South Bend, IN, USA). Male BALB/c mice were obtained from ORIENT (Gyeonggi‐do, Republic of Korea). LC–MS/MS grade solvents and analytical grade chemicals were obtained from J.T. Baker (Radnor, PA, USA) and SIGMA Aldrich (St. Louis, MO, USA), respectively.

### 2.2 In vivo brain uptake analysis

#### 2.2.1 Design of ICO device for mouse

**FIGURE 1:**
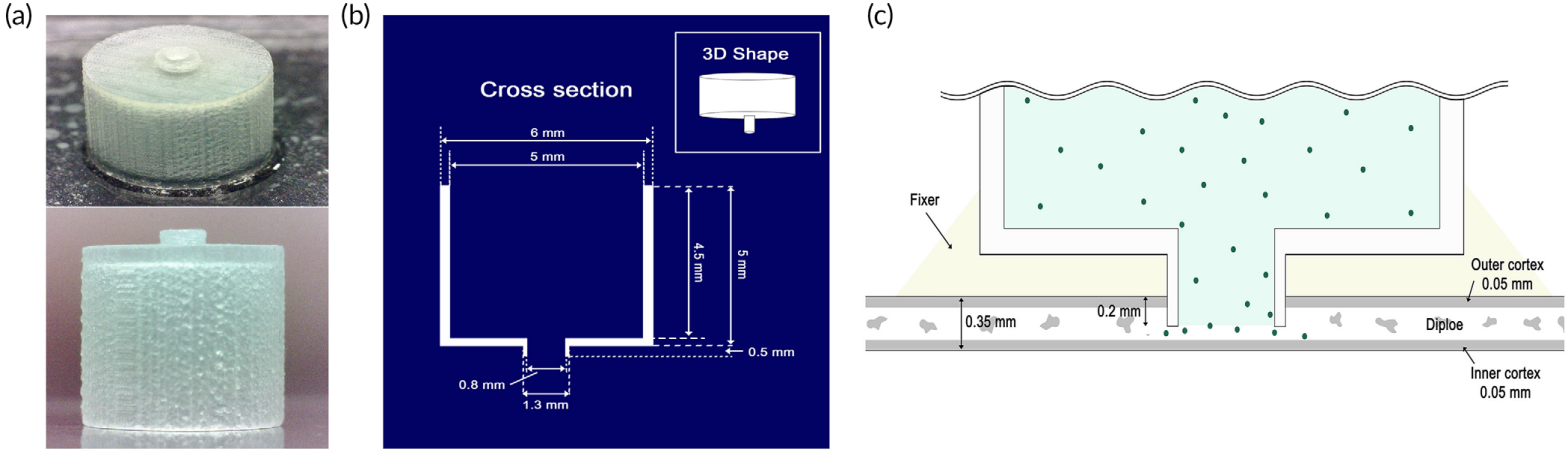
(a) Photos of real ICO device for the mouse. (b) Schematic cross section and 3D shape diagram of ICO device for mouse. (c) Schematic diagram of brain drug delivery through the ICO administration route with drug reservoir

A specific intracalvaiosseous administration device (ICO device), suitable for the mouse skull, was developed. A reservoir/skull‐embedding tube one‐body‐type ICO device (total height 5.5 mm) was designed by three‐dimensional (3D) printing technique (Figure 1a,b). The 3D printing material was USP Class VI certified VisiJet M3 Crystal (3D Systems, USA). The specifications of the skull embedding tube part are outer diameter (OD) 1.3 mm, inner diameter (ID) 0.8 mm, and height 0.5 mm. The skull embedding tube part of the ICO device was embedded at a 0.2 mm depth into the calvarial diploe (Figure 1b,c).

#### 2.2.2 Animals

All animal procedures were approved by the Institutional Animal Care and Use Committee at Gachon University, Republic of Korea, and complied with the guideline for users of Animal Ethics Committee of Gachon University (approval number LCDI‐2020‐0062). The experiment was conducted with male BALB/c mice aged 5 weeks with a weight range of 20–25 g. To ensure statistical independence, only male mice were used for each experiment. The animal experiments for nine compounds were conducted in series with IV and ICO group per compound as follows: (1) CPZ‐IV and ICO, (2) RIS‐IV and ICO, (3) DPZ‐IV and ICO, (4) RVG‐IV and ICO, (5) TMZ‐IV and ICO, (6) PTX‐IV and ICO, (7) GABA‐IV and ICO, (8) GSH‐IV and ICO, and (9) SUC‐IV and ICO. For each compound, eight mice were randomly divided into IV and ICO group (*n* = 4). All the experimental procedures were performed from the next day of randomization.

#### 2.2.3 Skull thinning for intraosseous administration into the skull (ICO)

To accomplish intraosseous administration into the skull (ICO administration) for evaluation of brain drug delivery, we performed skull thinning in mice. Mice were anesthetized with isoflurane and maintained at a core of 37°C. The left parietal cranial bone (−1.5 mm anterior/posterior (AP) and −1.5 mm medial/lateral (ML) from the bregma) over the outer cortex was thinned to a thickness of 200 μm (ventral from the skull surface), with a circle shape of 1.35 mm in diameter using the Robot stereotaxic system equipped with drill and injection Robot, and StereoDrive software (NeuroStar, Tubingen, Germany). The drilling for skull thinning was performed with a flat drill of 0.7 mm drill bit size (NeuroStar) on auto‐speed craniotomy‐shape mode by point‐by‐point edge cut. After thinning the skull, bone dust was cleared and then an ICO device was embedded in the thinned area of the skull and sealed by epoxy glue and dental cement. The skin wound was then sealed with sutures.

#### 2.2.4 Integrity test for ICO device embedded in thinned‐mouse skull by near‐infrared fluorescence imaging with indocyanine green

To test the integrity of ICO administration by ICO device embedding in a thinned‐mouse skull, we observed ICG diffusion into the mouse skull by near‐infrared (NIR) fluorescence imaging. 50 μl ICG (500 μg/ml in saline) was administered into the ICO device‐embedded mouse. After 24 h, the mice were placed on the stereotaxic with isoflurane inhalation anesthesia and then scalp was cut. For the ICG‐NIR fluorescence imaging, a 780‐nm laser with a power of 150 mW (LS Korea, Republic of Korea) was set up at a distance of 80 mm and an angle of ~20° from the target through the C‐clamp of Z stand for laser spot size with a width of 15 mm and a length of 25 mm. The fluorescence signal of ICG was directed from the imaging stage to the NIR camera equipped with an ICG filter (collection wavelength of 810–850 nm) (Electronic Auto System, Republic of Korea). We recorded fluorescence images using Basedcam2 software at 1 frame per second while NIR light was administered via a 780‐nm laser.

#### 2.2.5 Implantation of guide cannula for microdialysis probe

To implant microdialysis guide cannula, the mice for ICO and IV groups were positioned on a stereotactic device (Harvard Apparatus, USA) using ear bar under 1–1.5% isoflurane anesthesia in N_2_O:O_2_ (70:30 vol%). A guide cannula was inserted into the right hippocampus (−2.0 mm anterior/posterior (AP), 1.5 mm medial/lateral (ML), and −2.0 mm dorsal/ventral from the bregma) after drilling a small hole in the skull. The guide cannula was fixed with dental cement and the skin wound was sealed with sutures. The animals were then returned to their cages for 1 day and allowed to recover from the surgery.

#### 2.2.6 Compound administration and brain microdialysate, plasma, and whole brain sampling

For stabilization before compound administration, microdialysis probes were perfused with artificial cerebrospinal fluid (aCSF) buffer at a flow rate of 0.5 μl/min for 1 h with the membrane of the microdialysis probe soaking in ethanol. Nine compounds (CPZ, RIS, DPZ, RVG, TMZ, PTX, GABA, GSH, and SUC) were intravenously injected into each IV group by tail vein at a dose of 0.5 mg/kg and administered to the skull in each ICO group at a dose of 5 mg/kg by filling the compound solutions in the ICO device with capping.

Perfusates from the microdialysis probes were collected every 30 min for 4 h after administration, at 8 and 24 h. Dialysate samples were stored at −80°C until the time of LC–MS/MS analysis. Blood samples (30 μl) were collected from the saphenous vein at 2, 5, 10, 30, 60, 120, 240, 480, and 1440 min after administration into 0.6 ml heparinized tubes. The blood samples were immediately centrifuged at 10,000 rpm for 10 min at 4°C to recover plasma. In case of TMZ, the collected blood samples after administration were immediately centrifuged, and then 10 μl of plasma was acidified with 2 μl of 0.1% formic acid. The brain was then excised 24 h post‐administration and homogenized using a Bioprep‐24 homogenizer (Bioand, Seoul, Korea) after adding PBS equivalent to the weight of each tissue sample. Plasma and homogenized brain tissue samples were stored at −80°C until LC–MS/MS analysis. The details of sample preparation for quantitative analysis and LC–MS/MS analysis are described in the Supporting Information.

### 2.3 Data analysis

To compare the results of the nine compounds (CPZ, RIS, SUC, TMZ, PTX, DPZ, RVG, GABA, and GSH) according to administration route, the compound concentrations in plasma and ISF dialysate samples were determined using an LC–MS/MS analysis and plotted over time. The area under the curve in plasma concentration‐time curves (AUC_plasma_) and brain ISF concentration‐time curves (AUC_ISF_) were determined by non‐compartmental analysis using WinNonlin 2.1 (Pharsight, Mountain View, CA). The compound concentration in the whole brain at 24 h (*C*_br_) was determined using LC–MS/MS and then corrected for the plasma volume of the corresponding organ using equation:

$$C_{\mathrm{br}}=C_{\mathrm{br},\text{quant},24h}-\left(V_{0}\times C_{\text{plasma},24h}\right),$$

where *C*_br,quant,24h_ = brain concentration (ng/g) at 24 h quantified by LC–MS/MS, *V*_0_ = plasma volume of the corresponding organ (μl/g), *C*_plasma,24h_ = plasma concentration (ng/ml) at 24 h. The following *V*_0_ value for the brain was chosen as 9.3 ± 1.1 μl/g.^19^

The extent of brain uptake after ICO administration as compared to IV administration was also expressed as brain availability (*F*_br_), analogous to systemic bioavailability (*F*), and calculated as a ratio of AUC_ISF,IV_ and AUC_ISF,ICO_ using the equation:

$$F_{\mathrm{br}}={\mathrm{AUC}}_{\mathrm{ISF},\mathrm{ICO}}/{\mathrm{AUC}}_{\mathrm{ISF},\mathrm{IV}},$$

where AUC_ISF,ICO_ and AUC_ISF,IV_ are the area under the curve to 24 h in the brain ISF‐time plot (AUC_ISF_) after ICO and IV administration, respectively.

### 2.4 Statistical analysis

All data are presented as mean ± SEM. Each value represents the mean of four separate experiments for each group. Significant differences were analyzed using a two‐sample Student's *t*‐test. Statistical significance was indicated by *p < 0.05 and **p < 0.01.

## 3 RESULTS

### 3.1 Fabrication of ICO device and animal preparation for ICO administration in mice

**FIGURE 2:**
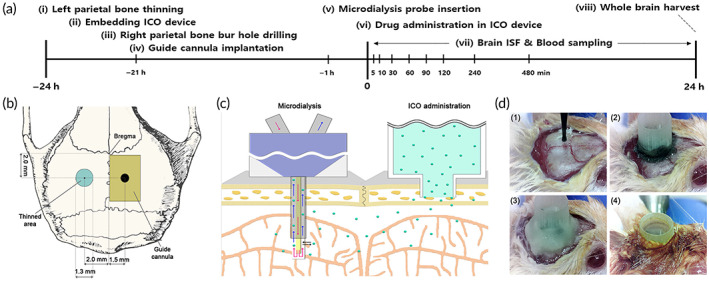
(a) Schematic diagram of the experimental design of the simulated ICO administration procedure and pharmacokinetic study schedule. (b) A schematic diagram of the mouse's dorsal skull surface showing the locations of the thinned skull area for ICO administration and the guide cannula for insertion of the microdialysis probe. (c) A schematic diagram of a mouse's coronal skull surface indicating the locations of an ICO device and an inserted microdialysis probe in the brain. (d) Actual photo of the ICO device embedding process, which includes (1) skull thinning, (2) ICO device inserting into the thinned skull area and epoxy glue fixing, (3) ICO device dental cement fixing, and (4) skin suturing

To actualize the ICO administration in the mice, we designed the ICO device for the mice (Figure 1a,b). ICO device is a one‐body‐type that consists of a cylindrical reservoir with a diameter of 5 mm and a skull‐embedding tube with an ID of 0.8 mm, OD of 1.3 mm, and a height of 0.5 mm. The light microscopy images (Figure S1) show that the ICO device was well fabricated with a flat surface with a surface roughness average of less than 25 μm enough to be seated into the thinned skull at 200 μm depth. It indicates that the ICO device is not inferior for embedding into the calvarial diploe, which is sponge cancellous bone, as 0.2 mm. The ICO device was sterilized in ethanol and applied to a thinned area on the mouse skull for ICO administration (Figure 1c). The flow chart for in vivo experimental design is shown in Figure 2a. The details of the thinned skull area and guide cannula place for insertion of microdialysis tube on the mouse skull were presented in Figure 2b by dorsal view. The left parietal cranial bone of the mouse was thinned to a thickness of 200 μm with a circle shape of 1.3 mm in diameter. The guide cannula for the insertion of the microdialysis tube was fixed by cement after drilling a bur hole at right parietal bone. The schematic diagram of microdialysis and ICO administration was presented in Figure 2c by coronal section view. Figure 2d shows a real mouse photo after embedding the ICO device.

### 3.2 Integrity test for ICO device embedded in thinned‐mouse skull by NIR fluorescence imaging with ICG

**FIGURE 3:**
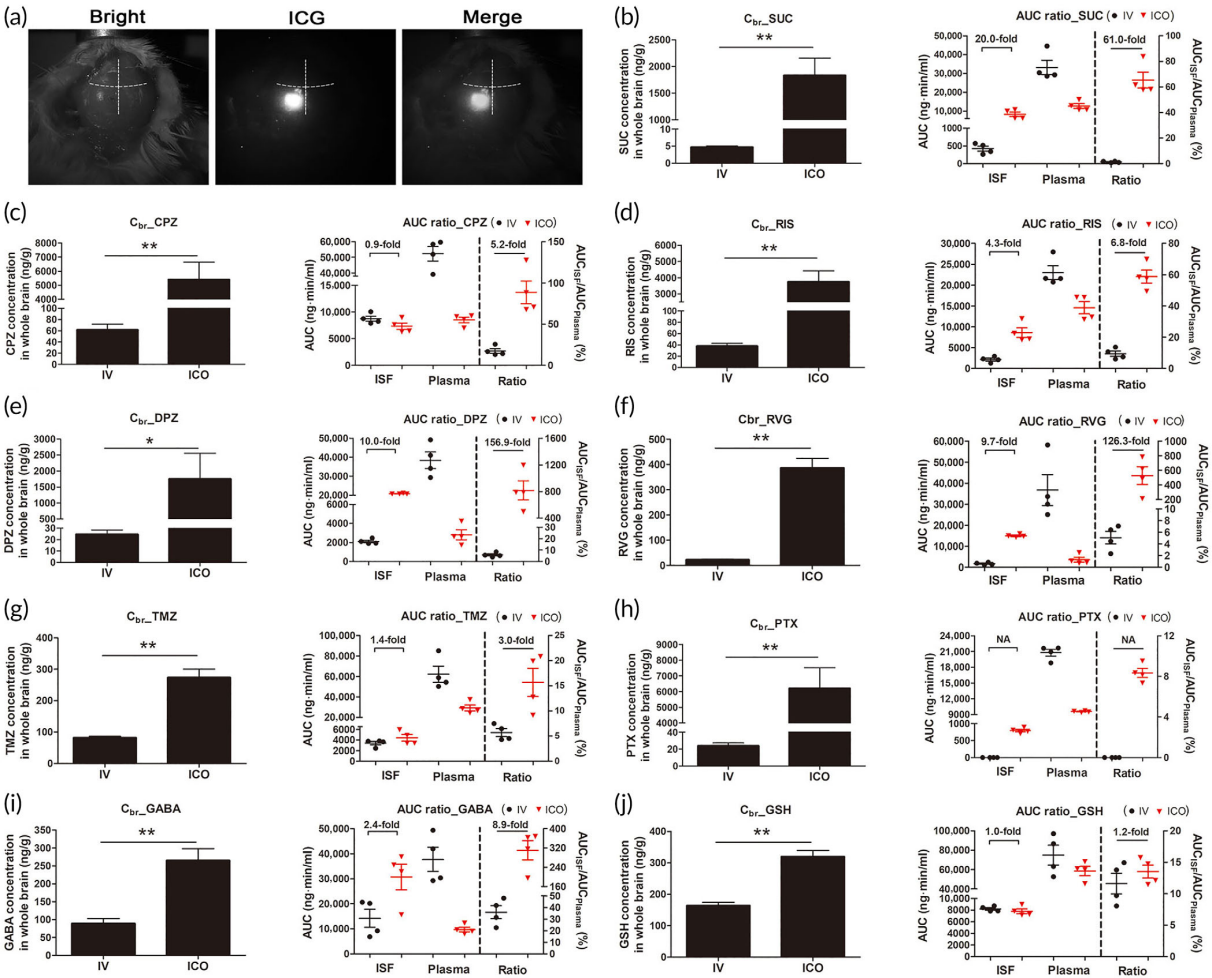
(a) NIR camera images after ICG diffusion inside only the left parietal bone after 24 h post‐ICO administration. The border line for showing each different bone was presented by white dot line based on the coronal suture and sagittal suture. (b) Compound concentration in whole brain at 24 h (*C*_br_) and AUC ratio after ICO and IV administration of sucrose (SUC), (c) chlorpromazine (CPZ), (d) risperidone (RIS), (e) donepezil (DPZ), (f) rivastigmine (RVG), (g) temozolomide (TMZ), (h) paclitaxel (PTX), (i) γ‐aminobutyric acid‐d6 (GABA) and (j) glutathione (GSH) (*n* = 4)

To verify the integrity of ICO administration, we applied indocyanine green (ICG) into the ICO device embedded in a thinned‐mouse skull. When we observed ICG fluorescence signals in the skull with an NIR camera after 24 h post‐ICO administration, the ICG signals shone brightly at the left parietal bone where the ICO device was embedded, whereas no signal was observed at the other skull bones. In addition, no leak from the glue‐sealed area was observed (Figure 3a). This clearly indicated that the ICG was diffused into the diploic space of the left parietal bone, where the ICO device was embedded.

It was estimated that the extent of drug partitioning from the ICO device to diploe within a 24 h period was approximately 10% of the device‐loading dose from the integrity test with ICG (Figure S2) and then 10‐times of the IV dose was applied to the ICO in further pharmacokinetic study.

### 3.3 Pharmacokinetic and in vivo brain uptake study after IV and ICO administration

To evaluate the comparative advantage for brain drug delivery of the ICO over the systemic administration route, we carried out quantitative pharmacokinetic analysis with eight CNS treatment‐related compounds and a control.

The plasma and ISF concentration‐time profiles of all the compounds and control after IV and ICO administration are presented in the Supporting Information (Figures S3–S11). The area under the curve of plasma‐time profiles (AUC_plasma_) and ISF‐time profiles (AUC_ISF_) and the ratio of AUC_ISF_ to AUC_plasma_ were calculated for each mouse. Whole brain tissue concentration at 24 h (*C*_br_), the AUC values, and the ratio of AUC_ISF_ to AUC_plasma_ are presented in Figure 3b–j. Table 1 summarizes the pharmacokinetic parameters of nine compounds after IV and ICO administration, including AUC_plasma_, AUC_ISF_, *C*_br_, brain/plasma ratio at 24 h (*K*_p_), and in vivo brain‐availability (*F*_br_).

**TABLE 1:** Pharmacokinetic parameter and in vivo brain‐availability (*F*_br_) of nine compounds after IV and ICO administration (mean ± SEM, *n* = 4)

Compound	Administration route	AUC_plasma_ (ng/min/ml)	AUC_ISF_ (ng/min/ml)	*C*_br_ (ng/ml)	*K*_p_ (*C*_br_/*C*_plasma_,_24h_)	*F*_br_ (AUC_ISF,ICO_/AUC_ISF,IV_)
CPZ	IV	56,290.00 ± 6265.71	8476.25 ± 476.68	61.46 ± 10.49	36.30 ± 8.27	0.85 ± 0.10
	ICO	8522.75 ± 519.72	7336.25 ± 616.98	5407.19 ± 1238.35	1280.14 ± 296.92	
RIS	IV	22,986.75 ± 1658.30	2148.50 ± 349.22	37.97 ± 4.82	21.89 ± 8.07	4.28 ± 0.69
	ICO	14,556.25 ± 1457.2	8598.00 ± 1167.7	3747.84 ± 678.6	794.58 ± 209.82	
DPZ	IV	38,440.75 ± 4315.11	2106.25 ± 133.99	24.63 ± 3.83	3.60 ± 0.42	9.99 ± 0.55
	ICO	2812.00 ± 525.02	20,816.75 ± 98.22	1754.76 ± 801.83	1122.15 ± 666.14	
RVG	IV	36,738.75 ± 7352.63	1695.75 ± 284.54	22.43 ± 2.43	2.25 ± 0.33	9.73 ± 1.82
	ICO	3605.00 ± 1167.39	14,989.25 ± 359.33	386.54 ± 37.65	242.46 ± 80.58	
TMZ	IV	62,153.00 ± 7855.31	3417.00 ± 324.97	81.70 ± 9.48	8.88 ± 0.53	1.35 ± 0.26
	ICO	29,226.25 ± 2830.94	4404.00 ± 615.08	273.84 ± 26.82	16.83 ± 1.60	
PTX	IV	20,763.00 ± 665.40	NA	23.78 ± 3.34	3.11 ± 0.49	NA
	ICO	9566.25 ± 73.39	796.55 ± 34.49	6801.40 ± 1231.95	1012.79 ± 178.66	
GABA	IV	37,720.25 ± 4821.54	14,212.00 ± 3596.81	89.19 ± 13.54	28.42 ± 9.22	2.41 ± 0.50
	ICO	9745.75 ± 911.87	30,689.75 ± 5158.23	264.93 ± 33.37	95.41 ± 36.02	
GSH	IV	74,953.50 ± 10,254.19	8212.75 ± 217.78	164.25 ± 10.13	5.66 ± 1.38	0.95 ± 0.04
	ICO	58,638.50 ± 4743.71	7811.50 ± 415.28	319.65 ± 19.72	8.09 ± 0.27	
SUC	IV	33,180.00 ± 3812.80	422.50 ± 71.28	5.00 ± 0.33	3.05 ± 1.32	20.01 ± 1.22
	ICO	12,616.50 ± 1264.01	8292.25 ± 1194.64	1724.72 ± 329.49	294.18 ± 169.79	

Statistical analysis of seven compounds (SUC, RIS, DPZ, RVG, TMZ, PTX, and GABA) revealed the same tendency that AUC_plasma_ of ICO groups was relatively lower than IV groups whereas AUC_ISF_ of ICO groups was relatively higher than IV groups. CPZ and GSH showed that AUC_plasma_ of ICO groups was relatively lower than IV groups whereas AUC_ISF_ of ICO groups was no different from IV groups. The AUC ratio (%, AUC_ISF_/AUC_plasma_) revealed that ICO groups were significantly higher than IV groups, ranging from 3.0‐fold (TMZ) to 156.9‐fold (DPZ), except PTX and GSH (Figure 3b–j). The brain availability (*F*_br_), analogous to systemic bioavailability (*F*), was calculated as a ratio of AUC_ISF,IV_ and AUC_ISF,ICO_ and is represented in Table 1. In particular, in the case of PTX, ISF concentration at all‐time points after IV administration was under the limit of detection (LOD), resulting in to be not analyzed (NA), whereas that after ICO administration was detected, resulting in to be calculated as an AUC_ISF_ of 796.55 ng/min/ml (Figure 3h). It is meaningful from the point of view that PTX, which could not sufficiently enter the brain to quantify after systemic administration, could do that after ICO administration.

Interestingly, the Cbr, which is a parameter representing actual brain drug concentration at 24 h, in all compounds showed that ICO groups were significantly higher than IV groups by as much as 85.1‐fold (CPZ), 115.2‐fold (RIS), 64.3‐fold (DPZ), 18.6‐fold (RVG), 3.4‐fold (TMZ), 294.1‐fold (PTX), 3.1‐fold (GABA), 3.4‐fold (GSH), and 342.0‐fold (SUC). These results supported our hypothesis that ICO administration may provide a special environment in which compounds can enter the brain.

In addition, brain to plasma ratio at 24 h (K_p_) in all compounds showed that ICO groups were significantly higher than IV groups by as much as 35.3‐fold (CPZ), 36.3‐fold (RIS), 311.3‐fold (DPZ), 107.8‐fold (RVG), 1.9‐fold (TMZ), 325.7‐fold (PTX), 3.4‐fold (GABA), 1.4‐fold (GSH), and 96.5‐fold (SUC). This indicates that brain accumulation following ICO administration might be handled differently compared to IV administration, which relies only on the BBB passing route for brain drug delivery.

### 3.4 Relationship between the in vivo brain uptake of compounds versus in vitro BBB permeability, molecular weight, and octanol–water partition coefficients

To investigate the relationship between the in vivo brain uptake of compounds and molecular characteristics, we evaluated the in vitro brain permeability of chosen compounds (Figure S12). CPZ, RIS, DPZ, RVG, and TMZ selected as representatives among CNS drugs showed a PS value of 10 or higher, indicating that they can be permeable to the brain compared to the control SUC which is representative of brain impermeable compound. In particular, GABA, a neurotransmitter and biological product for neuroprotective, showed the highest PS value of 26.89 ± 1.89, whereas PTX, a widely used anticancer agent but not used for brain tumors, showed the lowest PS value of 1.74 ± 0.04.

**FIGURE 4:**
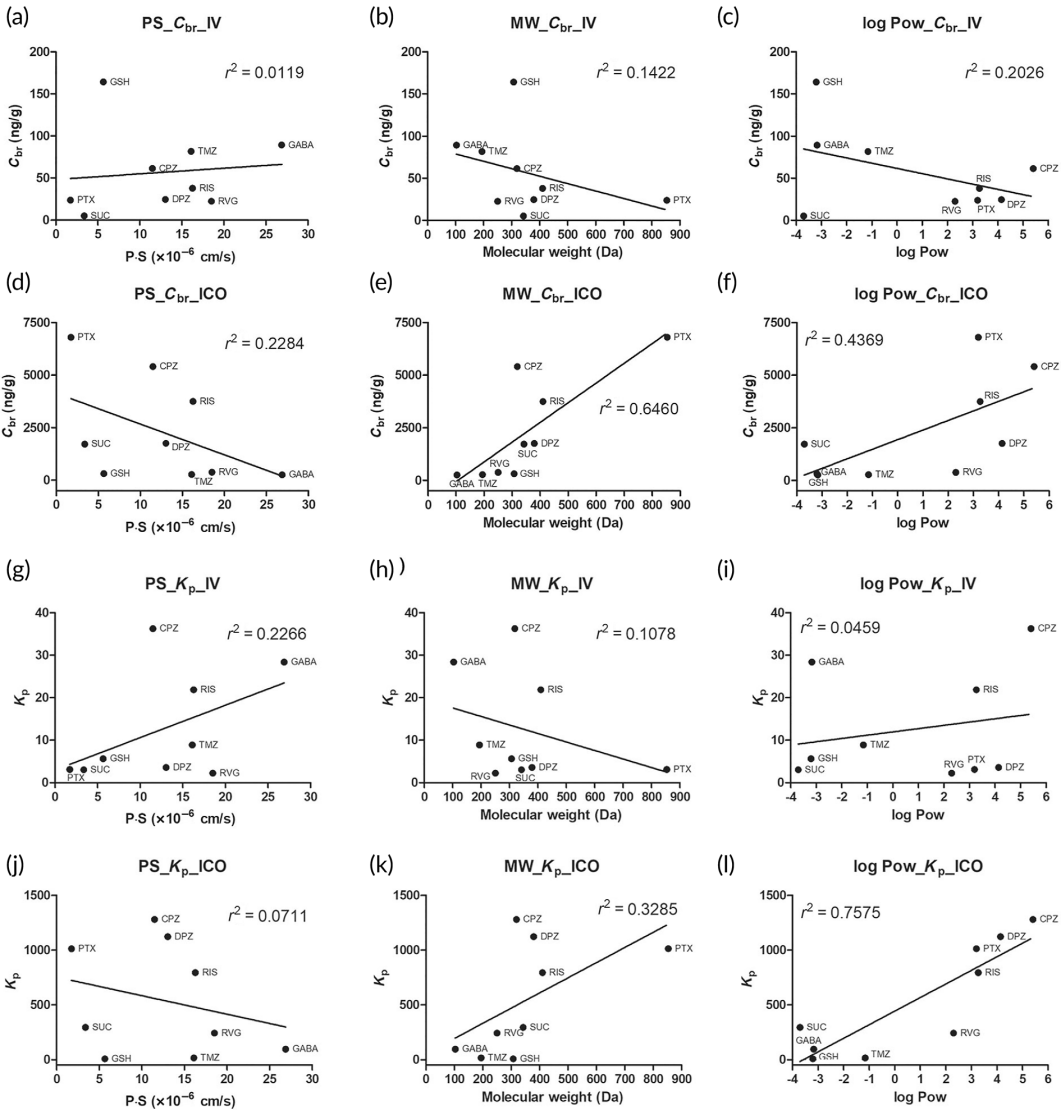
Relationship between experimental compound concentration in brain at 24 h (*C*_br_) after intravenous administration (IV) and experimental in vitro BBB permeability product‐surface area (*PS*) values (a), their molecular weight (MW) (b), and octanol–water partition coefficient (log Pow) (c). Relationship of *C*_br_ after intracalvariosseous administration (ICO) with the experimental in vitro PS values (d), MW (e), and log Pow (f). The relationship of calculated ratio of brain/plasma concentration at 24 h (*K*_p_) after IV with the experimental in vitro *PS* values (g), MW (h), and log Pow (i). The relationship of *K*_p_ after ICO with the experimental *PS* values (j), MW (k), and log Pow (l). The data are the average of four cell monolayers and four animals for each tested compound.

Table S2 summarizes the values of the experimental in vitro BBB permeability‐surface area (PS), molecular weight (MW), and octanol–water partition coefficients (log Pow). In addition, the PS, MW, and log Pow were plotted against experimental compound concentration in the brain at 24 h (*C*_br_) or the calculated ratio of brain/plasma concentration at 24 h (*K*_p_) after IV and ICO administration for over nine compounds (Figure 4). The lines show the relationship between the in vivo brain uptake values and each parameter (PS, MW, and log Pow) for the nine compounds. In group IV, the higher the PS values, the higher the *C*_br_, whereas the higher the MW and log Pow, the lower the *C*_br_ (Figure 4a–c). In contrast, in the case of the ICO group, the lower the PS value, the higher the *C*_br_, whereas the lower the MW and log Pow, the lower the *C*_br_ (Figure 4d–f). Although no distinct trend was observed with r square values (0.0119–0.6460), the regression line between the IV and ICO groups showed a clearly opposite slope direction. Likewise, the regression lines of the IV and ICO groups showed clearly opposite slopes in the relationship between Kp, PS, and MW (Figure 4g,h,j,k). In the case of the relationship between Kp and log Pow, as shown in Figure 4i,l, the slope direction of the regression line between group IV and ICO was the same, but the relationship degree was clearly distinguished by *r* ^2^ (IV: 0.0587 and ICO: 0.7381). These results indicated that the brain uptake of compounds after ICO administration would be processed in different ways, resulting in higher brain exposure and accumulation of compounds compared to IV administration.

## 4 DISCUSSION

In this study, our data suggest that intraosseous administration into the skull (ICO) can be a novel approach for brain drug delivery of CNS drugs via BBB‐bypassing routes. Importantly, the brain concentration of the nine representative compounds after ICO administration was higher (minimum 2‐fold to maximum 342‐fold) than that after IV administration, suggesting that ICO administration might provide a special condition in which compounds have an opportunity to be taken up into the brain.

**FIGURE 5:**
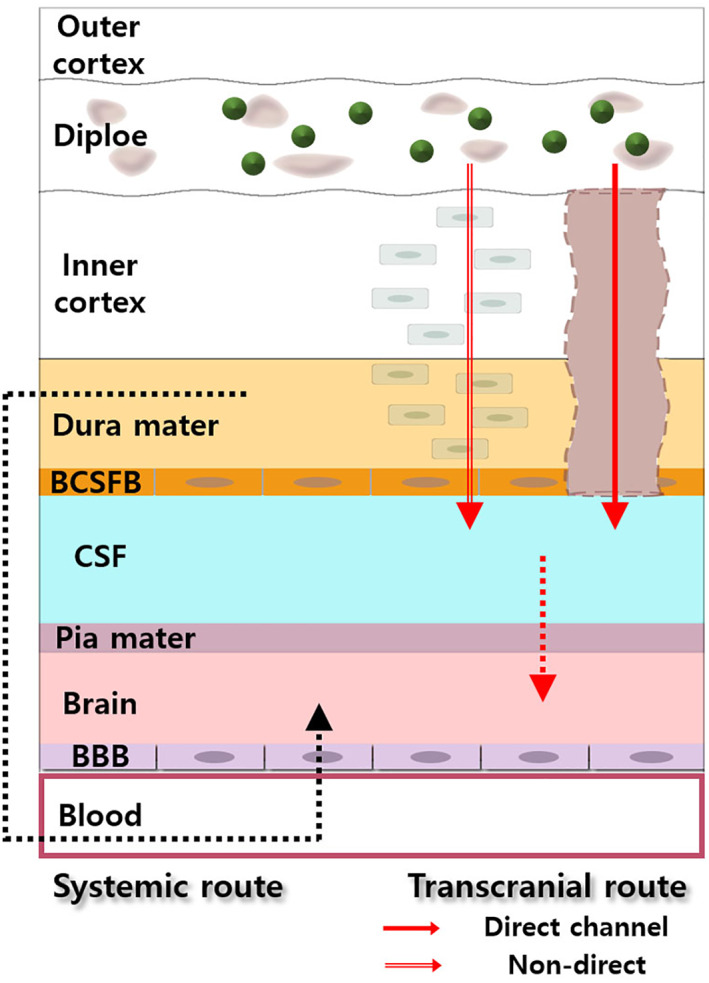
The key transcranial route of molecules from the skull cortex penetration to the brain following ICO administration. Molecules are transported to the brain through not only the systemic route but also the transcranial route involving direct channel and non‐direct.

In our findings, the regression line in the correlation analysis showed an opposite slope direction between ICO and IV administration in the whole brain concentration (*C*_br_) and brain/plasma ratio at 24 h (*K*_p_) related to molecular characteristics (Figure 4). In the case of IV injection directly into the blood stream, the drugs should pass through the BBB to enter the brain, resulting in the brain concentration being influenced by the blood concentration of drugs. It has been reported that hydrophilic compounds have limited passive transport of drug molecules through the BBB, whereas lipophilic drugs smaller than 400–600 Da may enter the brain with comparative ease.^20^ This is consistent with our results in the IV correlation data, showing that the lower the PS values, the lower the *C*_br_, whereas a lower MW and log Pow lead to a higher *C*_br_. However, in the case of ICO administration into the skull diploe, which showed opposite results to those with IV administration, it can be expected that the brain uptake of compounds after ICO administration is processed through a different route compared to the systemic route, which is only related to the blood‐to‐brain route. We expected that the transportation of molecules from the diploe to the brain would occur through the above‐mentioned transcranial route. The skull‐to‐brain (transcranial) route may involve direct channels and non‐direct pathways (Figure 5).

The meninges consist of three layers, including the dura mater, the arachnoid mater, and pia mater, that surround the brain and spinal cord.^21^, ^22^ The arachnoid barrier cell layer is distinguished by continuous and complex tight junctions, forming an epithelial blood‐cerebrospinal fluid (BCSFB) between the dura mater and the subarachnoid space.^21^, ^22^, ^23^ The CSF was filled in the subarachnoid space below the arachnoid barrier. The pia mater is a thin and transparent layer for CSF separation from the brain.^24^ The pia mater has no tight junctions,^21^ thus molecules can get the opportunity to easily enter the brain if they reach the CSF. It was reported that CSF accesses skull bone marrow via direct channels.^17^ These channels could enable bone marrow's direct access to the CSF, allowing a route for drug delivery from the skull to the brain. Although the meninges, providing a CNS barrier, might be one impediment to the transport of drugs from the skull to the brain by ICO administration, our results strongly showed that the skull is not completely impermeable to drug penetration into the brain. The details of the drug delivery process by ICO through the transcranial route are currently unknown; it would be an interesting study to closely explore the function of the direct channel in view of the drug delivery mechanism.

To administer BBB‐impermeable CNS drugs for the treatment of brain disease, recently marketed biologics have chosen local direct invasive administrations as BBB‐bypassing alternative routes, including surgical implants (intracerebroventricular [ICV]), or spinal cavity (intrathecal [IT]).^25^ These offer the possibility of reaching higher local drug concentrations in the appropriate target regions while minimizing those in other brain or peripheral areas compared with those via the systemic route.^26^, ^27^ Nevertheless, the approaches still have limitations, such as invasive procedures and associated complications.^28^, ^29^, ^30^ In light of these limitations related to current attempts for brain delivery of BBB‐impermeable CNS drugs, ICO administration could be a potential alternative technique for brain drug delivery to address these concerns.

This study presents a new administration technique using the skull for brain drug delivery in mice. The parietal bone, which is the largest in the mouse skull (length 4.8 mm, width^31^ and approximate skull thickness of 340 μm^32^) was chosen for ICO administration. The test compounds were selected from the widely used CNS drugs in four clusters, with a wide range of molecular properties: CPZ and RIS, the first and second antipsychotics; DPZ and RVG, Alzheimer's drugs; TMZ and PTX, anti‐cancer agents; GABA and GSH, biological products for neuroprotective. Additionally, sucrose, which is a marker of BBB impermeability, was used as a control. The ICO device embedding and compound administration processes were carefully conducted to avoid contamination from another route for the brain delivery of compounds, resulting in successful performance to verify the feasibility of ICO administration in mice. Nevertheless, this process should be further optimized to better simulate clinical conditions, including scale‐up to large animals, improvement and development of ICO devices, and addressing the expected complications.

The present study was focused only on short‐term pharmacokinetic studies within 24 h after a single dose. The compound adsorption—how long it takes for the compounds to be partitioned from the ICO device to the diploe and then permeated from the diploe to the brain parenchyma—of ICO was estimated from the integrity test with ICG. Even though the extent of adsorption may vary with compounds and the estimated value from the ICG should be used carefully, the difference is expected to be minimal under the employed experimental condition of 24 h. It should be noted that this study has a limitation of direct comparison between IV and ICO without the experimental determination on the extent of ICO absorption per compound. The absorption after ICO was slow and not complete for this experimental setting of 24 h and thus the decisive conclusions by comparing the plasma and/or tissue concentrations between the routes should be limited. Nevertheless, the AUC of brain intestinal fluid concentration after ICO was higher than those after IV for all the tested compounds, which would still be meaningful for the demonstration of the feasibility of ICO administration as a novel and systemic circulation non‐dependent route. To further compensate for the limitations of the short‐term pharmacokinetic studies, long‐term pharmacokinetic studies, including the kinetic profiles of the drug absorption, are ongoing in a chronic neurological disease.

The professional medical surgery under anesthesia—the skull thinning and device mounting—would be needed for clinical application of ICO but expected to be minimally invasive and a one‐time procedure. Although it is too early to provide any plausible predictions on the future of clinical application, ICO could find it more useful for the treatment of chronic neurological diseases than acute conditions such as strokes and head injuries. Furthermore, ICO could probably provide a less invasive alternative to the current IT devices after further optimization of many technical details.

Interestingly, in this study, we found that intraosseous administration into the skull readily delivered drugs into the brain, probably via the BBB‐bypassing route. This approach could be applied to other CNS drugs, particularly BBB‐impermeable drugs including nucleic acid therapeutics, peptides, and antibodies. Clinical applicability and ethical issues with the ICO should be carefully evaluated, although these were not within the scope of this study. Here, only the technical feasibility of ICO was proposed. Many technical details, such as appropriate devices, formulations, dosing regimens, and validated procedures, should be addressed prior to clinical application. Despite all the limitations, we believe this challenging study on the unprecedented ICO route could serve as a milestone in the area of brain drug delivery.

## AUTHOR CONTRIBUTIONS

**Ji Hee Kang:** Conceptualization (equal); data curation (equal); formal analysis (equal); funding acquisition (equal); investigation (equal); methodology (equal); resources (equal); validation (equal); visualization (equal); writing – original draft (equal); writing – review and editing (equal). **Young Tag Ko:** Conceptualization (equal); funding acquisition (equal); project administration (equal); supervision (equal); validation (equal); writing – original draft (equal); writing – review and editing (equal).

## CONFLICT OF INTEREST

The authors declare that they have no conflict of interest.

### PEER REVIEW

The peer review history for this article is available at https://publons.com/publon/10.1002/btm2.10424.

## Supporting information


**Appendix S1** Supporting Information.

## Data Availability

All data needed to evaluate the conclusions in the paper are present in the paper and/or the Supplementary Materials.
