# Supplementary material for: Intraosseous administration into the skull: Potential blood–brain barrier bypassing route for brain drug delivery
Source: Bioeng Transl Med. 2022 Oct 21;8(2):e10424. doi: 10.1002/btm2.10424 (PMC10013776; doi:10.1002/btm2.10424)
Supplement: Supplementary file 1 — Appendix S1 Supporting Information. [file BTM2-8-e10424-s001.docx]

**Supplementary Materials**

**Methods**

***In vitro* BBB permeability by BBB monolayer**

To evaluate the *in vitro* BBB permeability of nine compounds (CPZ, RIS, DPZ, RVG, TMZ, PTX, GABA, GSH, and SUC), we performed a transpenetration study using an *in vitro* BBB monolayer model. Brain endothelial bEnd.3 cells were seeded at a density of 3.0 x 10^4^ cells on Transwell permeable inserts (0.4 μm, 6.4 mm in diameter; Falcon^®^, Corning, NY, USA) with complete Dulbecco’s minimum Eagle’s medium (DMEM) and incubated at 37℃ in a 5% CO_2_ atmosphere for 5 days. The integrity of the BBB model was confirmed by transendothelial electrical resistance (TEER) measurements using EVOM2 (World Precision Instruments, Sarasota, FL, USA). When the TEER value was above 250 Ω, the Transwell insert was washed with PBS, and 100 μL of Ringer-Hepes buffer containing compound (20 μg/mL final concentration of compound in SFM) was added to the donor chamber. The receiver chamber was filled with 500 μL Ringer-HEPES buffer. The buffer with compounds from the receiver was collected, and the receiver part was refilled with fresh buffer at pre-determined times (10, 20, and 30 min). The collected samples were stored at -80°C until the time of LC-MS/MS analysis. After quantification of the compounds in the samples, the permeability-surface area product (PS), indicating the *in vitro* BBB permeability, was calculated using the following equation:

$$P\cdot S=(C_{receiver}\times V_{receiver})/(A\times t\times C_{donor})$$

where C_donor_, C_receiver_, V_receiver_, A, and t are the compound concentration in the donor, compound concentration in the receiver, receiver volume, membrane area, and incubation time, respectively.

**Estimation of the extent of drug partitioning from the ICO device to the diploe for 24 h**

To estimate the extent of ICG partitioning from the ICO device to diploe, we carried out ICG quantification by NIR fluorescence image analysis. 1 μL of the standard ICG samples (1 – 100 μg/mL) and before-after (24 h) ICO administered samples were dropped on the para film (15 mm x 25 mm). The fluorescence images were recorded using Basedcam2 software with 780 nm NIR light. The ICG concentration was quantified using image j software with the fluorescence images. The standard curve was used to calculate the concentration of ICG. The extent of drug partitioning from the ICO device to the diploe was calculated using the equation as follows:

$$Extent of drug partioning (\%)= \frac{C_{ICG,before}-C_{ICG,after}}{C_{ICG,before}}\times100$$

where, C_ICG,before_ = ICG concentration that was administered into the ICO device and C_ICG,after_ = ICG concentration that remained in the ICO device after 24 h.

***In vivo* assessment of microdialysis probe recovery**

A total of 6 kDa CMA 7 microdialysis probes (Havard apparatus, Holliston, MA, USA) were connected with PE/PVC tubing (0.6 ⅹ 1.6 mm) to a PHD ULTRA^TM^ syringe pump (Havard apparatus, Holliston, MA, USA). For equilibration of microdiaysis probe, the inlet of the microdialysis probe was perfused with filtered artificial cerebrospinal fluid (aCSF) buffer containing 122 mM NaCl, 1.3 mM CaCl_2_, 1.2 mM MgCl_2_, 3.0 mM KH_2_PO_4_, and 25.0 mM NaHCO_3_ at a flow rate of 0.5 μL/min, and the outlet was kept in empty Eptube for 1 h, with the membrane of the microdialysis probe soaking in ethanol. To estimate *in vivo* recovery, retrodialysis was performed with aCSF containing each compound (CPZ, RIS, DPZ, RVG, TMZ, PTX, GABA, GSH, and SUC) [^1^](#_ENREF_1). The *in vivo* recovery was calculated as equivalent to the loss upon retrodialysis using the following equation:

*In vivo* recovery $=\frac{C_{perfusate} - C_{dialysate}}{C_{perfusate}}$

where C_perfusate_ is the compound concentration in the perfusate (inlet of the microdialysis probe), and C_diaysate_ is the compound concentration in the dialysate (outlet of the microdialysis probe). Recovery was utilized to calculate the C_ISF_, which is the concentration of the compound in the brain ISF, from the measured brain ISF dialysate concentration [^2^](#_ENREF_2).

**Quantitative analysis of compounds in dialysate, plasma and whole brain samples**

***In vitro* BBB monolayer permeability samples**

An 10 μL aliquot of BBB monolayer permeability sample was mixed with a mobile phase containing an internal standard of 10 μL by vortexing for 1 min. In case of TMZ, an aliquot of 10 μL of BBB monolayer permeability sample was mixed with 0.1% FA acetonitrile containing internal standard of 20 μL by vortexing for 1 min. In the case of GSH, an aliquot of 10 μL of BBB monolayer permeability sample was mixed with 10 mM dithiothreitol (DTT) (10 μL) and a mobile phase containing an internal standard of 10 μL by vortexing for 1 min. After centrifugation at 10,000 rpm at 4°C for 10 min, 2 μL of the supernatant was immediately analyzed using LC-MS/MS.

**Plasma and brain sample preparation**

**Chlorpromazine, risperidone, donepezil, rivastigmine and paclitaxel:** CPZ, RIS, DPZ, RVG, and PTX were isolated from plasma and whole-brain homogenates by liquid-liquid extraction (LLE). Plasma samples (10 μL) and brain homogenate samples (50 μL) were mixed with 10 μL of the mobile phase containing an internal standard by vortexing for 1 min. Then, 250 μL ethyl acetate was added to the mixture and vortexed for 1 min. After centrifugation at 10,000 rpm and 4 °C for 10 min, the organic layer was separated and evaporated to dryness under vacuum. Finally, the dried residue was reconstituted in 20 μL of mobile phase (acetonitrile and 0.01% formic acid-water, 50:50%, v/v) and immediately analyzed by LC-MS/MS.

**Temozolomide:** TMZ was isolated from plasma and whole-brain homogenates by protein precipitation. Acetonitrile with 0.1% formic acid (40 μL) containing internal standard was added to plasma samples of 10 μL. Similarly, 0.1% formic acid-acetonitrile of 200 μL was added to brain homogenate samples of 50 μL. The mixture was vortexed and centrifuged at 10,000 rpm at 4 °C for 10 min. The deproteinized supernatant was transferred to HPLC vials and immediately analyzed by LC-MS/MS.

**γ-Aminobutyric Acid-d6:** GABA was isolated from plasma and whole-brain homogenates by protein precipitation. Acetonitrile and water mixture (80:20%, v/v) of 90 μL containing ^13^C_6_Sucrose as internal standard was added to plasma samples of 10 μL. Similarly, 450 μL acetonitrile and water were added to 50 μL brain homogenate samples. The mixture was vortexed and centrifuged at 10,000 rpm at 4 °C for 10 min. The deproteinized supernatant was transferred to HPLC vials and immediately analyzed using LC-MS/MS.

**Glutathione (glycine-13C2, 15N):** GSH was isolated from plasma and whole-brain homogenates by protein precipitation. DTT (10 mM, 10 μL), mobile phase containing internal standard of 10 μL, and 15% trichloroacetic acid (TCA) of 30 μL, were added to 10 μL plasma samples. Similarly, 50 μL 10 mM DTT, mobile phase containing internal standard, and 10 μL of 15% TCA, 200 μL, were added to brain homogenate samples of 50 μL. The mixture was vortexed and centrifuged at 10,000 rpm at 4°C for 10 min. The deproteinized supernatant was transferred to HPLC vials and immediately analyzed using LC-MS/MS.

**Brain ISF dialysate sample preparation**

An aliquot of 10 μL of brain ISF dialysate sample was mixed with a mobile phase containing an internal standard of 10 μL by vortexing for 1 min. In case of TMZ, an aliquot of 10 μL of brain ISF dialysate sample was mixed with 0.1% FA acetonitrile containing internal standard of 20 μL by vortexing for 1 min. For GSH, an aliquot of 10 μL of brain ISF dialysate sample was mixed with 10 mM DTT (10 μL) and a mobile phase containing an internal standard (10 μL) by vortexing for 1 min. After centrifugation at 10,000 rpm at 4°C for 10 min, 2 μL of the supernatant was immediately analyzed using LC-MS/MS.

**^13^C_12_Sucrose:** SUC was isolated from plasma and whole-brain homogenates by protein precipitation. Acetonitrile and 0.1% formic acid-water mixture (80:20%, v/v) of 90 μL containing ^13^C_6_Sucrose as internal standard was added to plasma samples of 10 μL. Similarly, 450 μL acetonitrile and water were added to 50 μL brain homogenate samples. The mixture was vortexed and centrifuged at 10,000 rpm at 4°C for 10 min. The deproteinized supernatant was transferred to HPLC vials and immediately analyzed by LC-MS/MS.

**LC-MS/MS analysis**

An Agilent 6490 triple quadrupole mass spectrometer (MS) (Agilent Technologies, CA, USA) with an electrospray ionization (ESI) source was equipped with an Agilent LC 1100 series binary pump, vacuum degasser, and auto-sampler system. MS/MS analyses were performed in positive ESI mode, and the target compounds were identified using multiple reaction monitoring (MRM) mode. The detailed MS parameters were as follows: collision gas, argon; capillary voltage, 5 kV; gas temperature, 225℃; gas flow at 15.1 L/min, and nebulizer pressure, 40 psi. All the columns were used at 45℃ under each condition of mobile phase, at a flow rate of 0.10 mL/min.

**Table S1.** MRM transition and optimized parameters of LC-MS/MS for nine compounds.

| Compound | Column | Mobile phase condition  (A: 0.1% formic acid in water  B: acetonitrile) | Transition  (positive mode) | Collision energy |
| --- | --- | --- | --- | --- |
| Chlorpromazine | Sepax BR-C18 (5 μm, 120 Å 1.0 × 100 mm) | Isocratic (A:B = 40:60, v/v)  0.10 mL/min | 319.1-57.8 | 30 eV |
| Risperidone | Sepax BR-C18 (5 μm, 1.0 × 100 mm) | Isocratic (A:B = 40:60, v/v)  0.10 mL/min | 411.0-191.1 | 24 eV |
| Donepezil | Sepax BR-C18 (5 μm, 1.0 × 100 mm) | Isocratic (A:B = 40:60, v/v)  0.10 mL/min | 380.1-91.1 | 36 eV |
| Rivastigmine | Sepax BR-C18 (5 μm, 1.0 × 100 mm) | Isocratic (A:B = 40:60, v/v)  0.10 mL/min | 251.2-206.1 | 30 eV |
| Temozolomide | ZORBAX Extend-C18 (3.5 μm, 1.0 × 50 mm) | Isocratic (A:B = 30:70, v/v)  0.10 mL/min | 195.0-98.0 | 6 eV |
| Paclitaxel | ZORBAX Extend-C18 (3.5 μm, 1.0 × 50 mm) | Isocratic (A:B = 30:70, v/v)  0.10 mL/min | 876.3-308.0 | 20 eV |
| γ-Aminobutyric Acid-d6 | ZORBAX Bonus-RP (3.5 μm, 1.0 × 50 mm) | A gradient  (5% B at 0 min, 5% B at 0.5 min, 95% B at 2.5 min, 95% B at  3 min, 5% B at 4 min, 5% B at 5 min)  0.10 mL/min | 110.0-93.0 | 8 eV |
| Glutathione (glycine-13C2, 15N) | ZORBAX Bonus-RP (3.5 μm, 1.0 × 50 mm) | A gradient  (5% B at 0 min, 5% B at 0.5 min, 95% B at 2.5 min, 95% B at  3 min, 5% B at 4 min, 5% B at 5 min)  0.10 mL/min | 311.1-75.8 | 24 eV |
| ^13^C_12_Sucrose | Waters ACQUITY BEH Amide (1.7 μm, 1.0 × 50 mm) | A gradient  (70% B at 0 min, 20% B at 2.5 min, 20% B at 3 min, 70% B at  4.5 min, 70% B at 5.5 min)  0.10 mL/min | 377.1-209.0 | 24 eV |

**Chlorpromazine, risperidone, donepezil and rivastigmine:** To separate the chromatographic compounds, an analytical Sepax BR-C18 (5 μm, 1.0 × 100 mm) column was used at 45℃ under isocratic conditions of mobile phase at a flow rate of 0.10 mL/min. The analytes were eluted using acetonitrile and 0.1% formic acid in water (60:40, v/v) and the isocratic separation run time was set to 5 min. The analytes were detected using the MRM mode to monitor the precursor-to-product ion transitions of 319.1-57.8. CPZ, 411.0-191.1. RIS, 380.1-91.1. DPZ, 251.2-206.1 for RVG and 415.1–195.1 m/z for RIS-d4 with collision energies of 30, 24, 36, 30, and 34, respectively.

**Temozolomide and paclitaxel:** To separate the chromatographic compounds, analytical ZORBAX Extend-C18 (5 μm, 1.0 × 50 mm) column was used at 45℃ under isocratic conditions of mobile phase at a flow rate of 0.10 mL/min. The analytes were eluted using acetonitrile and 0.1% formic acid in water (70:30, v/v) and the isocratic separation run time was set to 5 min. The analytes were detected using the MRM mode to monitor the precursor-to-product ion transitions of 195.0-98.0 for TMZ and 876.3-308.0 for PTX with collision energies of 6 and 20, respectively.

**γ-Aminobutyric Acid-d6 and Glutathione (glycine-13C2, 15N):** To separate the chromatographic compounds, analytical ZORBAX Bonus-RP (3.5 μm, 1 mm x 50 mm) column was used at 45℃ under isocratic conditions of mobile phase at a flow rate of 0.10 mL/min. The analytes were eluted using acetonitrile and 0.1% formic acid in water (5:95, v/v) and the gradient elution profile consisted of a 0.5–2.5 min linear gradient from 5% acetonitrile to 95% acetonitrile, holding 30 s. The column was re-equilibrated with 5% acetonitrile for 1 min between analyses, holding 1 min. The analytes were detected using the MRM mode to monitor the precursor-to-product ion transitions of 110.0-93.0, GABA-d6 and 311.1-75.8 for GSH (glycine-13C2, 15N) with collision energies of 8 and 24, respectively.

**^13^C_12_Sucrose:** To separate the chromatographic compounds, the analytical Waters ACQUITY BEH amide (1.7 μm, 1 mm × 50 mm) column was used at 45℃ under isocratic conditions of mobile phase at a flow rate of 0.10 mL/min. The analytes were eluted using acetonitrile and 0.1% formic acid in water (70:30, v/v), and the gradient elution profile consisted of a 2.5 min linear gradient from 70% acetonitrile to 20% acetonitrile, holding 30 s. The column was re-equilibrated with 70% acetonitrile for 2 min between analyses, holding 1 min. The analytes were detected using the MRM mode to monitor the precursor-to-product ion transitions of 377.1-209.0 for ^13^C_12_Sucrose and 371.0-203.0 for ^13^C_6_Sucrose with collision energies of 24 and 20, respectively.

**Supplementary Figures**


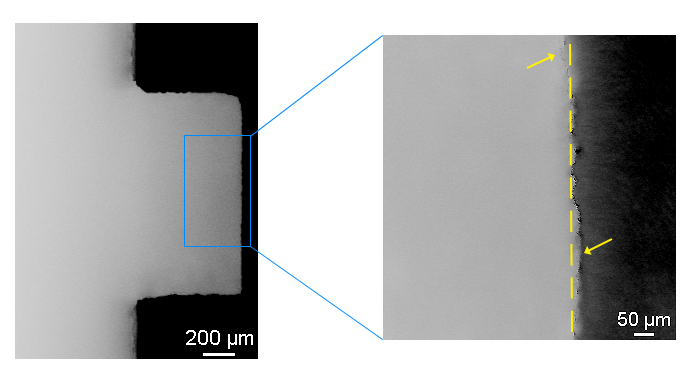


**Fig. S1.** Light microcopy imaging of cannulated tube part of ICO device. The color of images was inverted. The surface roughness of ICO device was visually estimated by the light microscscope images. The yellow dot-line indicates the mean line of surface roughness. The yellow arrows indicate the maximum peak and valley from mean line.


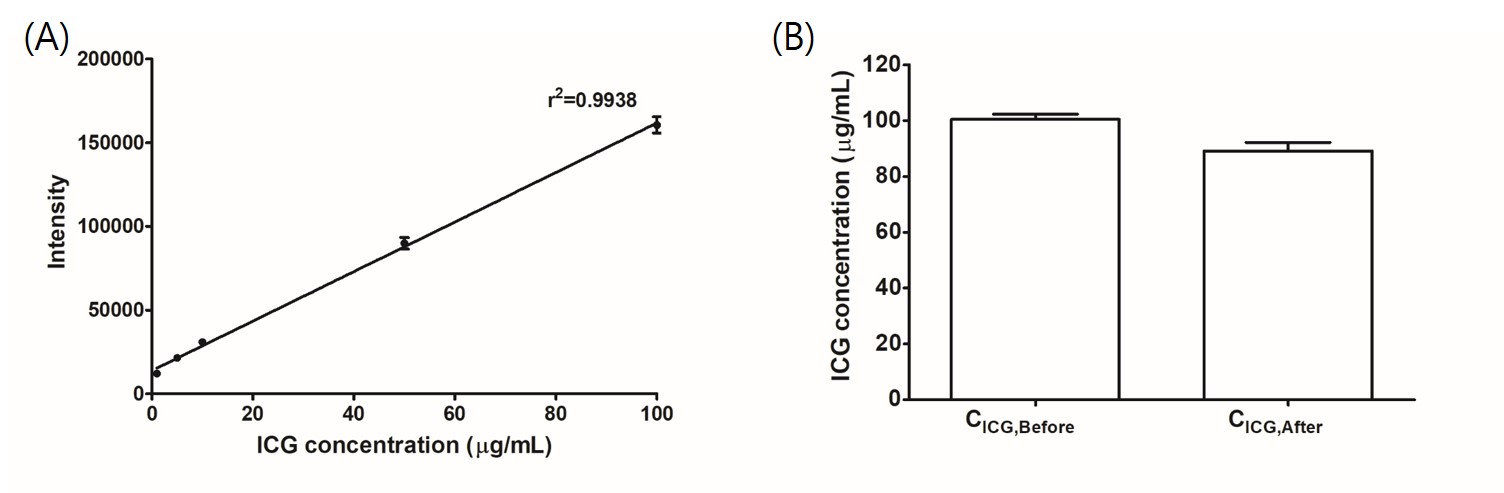


**Fig. S2.** Estimation of the extent of drug partitioning from the ICO device to the diploe for 24 h by quantification of ICG concentration using NIR-fluorescence image. (A) Standard calibration curve for determining ICG concentration. (B) The quantified ICG concentration that was administered into the ICO device (C_ICG,before_) and remained in the ICO device after 24 h (C_ICG,after_) (n=3). The extent of ICG partitioning was calculated as approximately 10% using the equation.


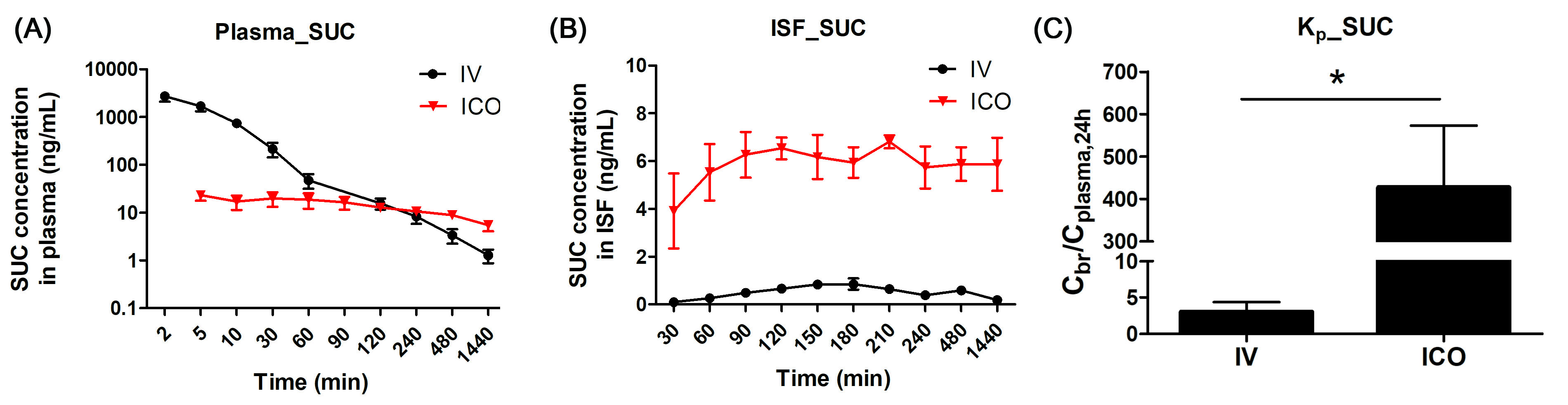


**Fig. S3.** The concentration-time profiles of sucrose (SUC) in the plasma (A) and ISF (B) following a single intravenous (IV) and intracalvariosseous (ICO) administration in mice. (C) Calculated ratio of brain/plasma concentration at 24 h (K_p_) after IV and ICO administration of SUC (mean±SEM, n=4).


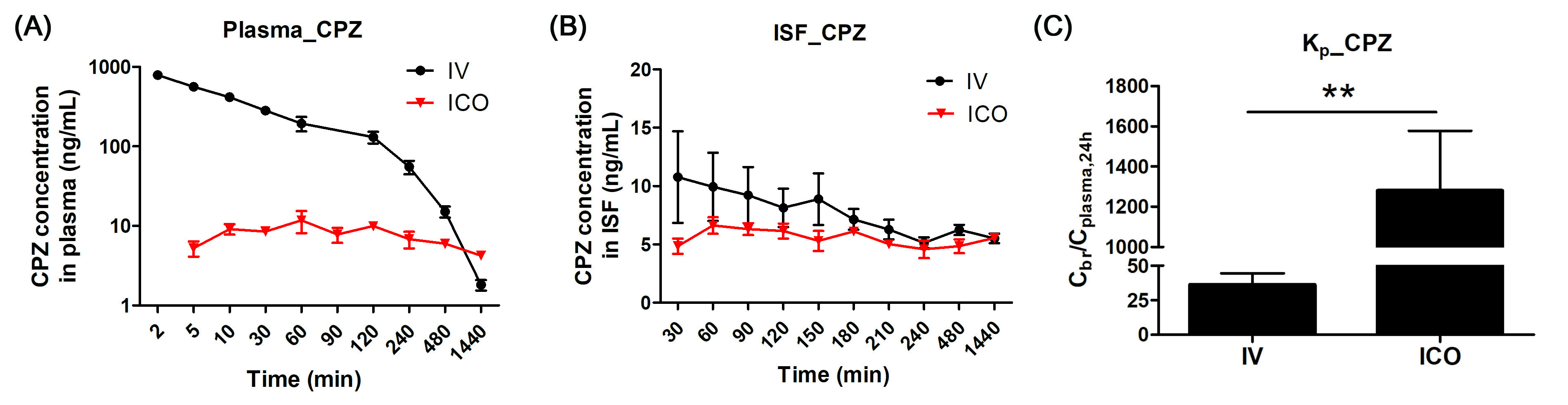


**Fig. S4.** The concentration-time profiles of chlorpromazine (CPZ) in the plasma (A) and ISF (B) following a single intravenous (IV) and intracalvariosseous (ICO) administration in mice. (C) Calculated ratio of brain/plasma concentration at 24 h (K_p_) after IV and ICO administration of CPZ (mean±SEM, n=4).


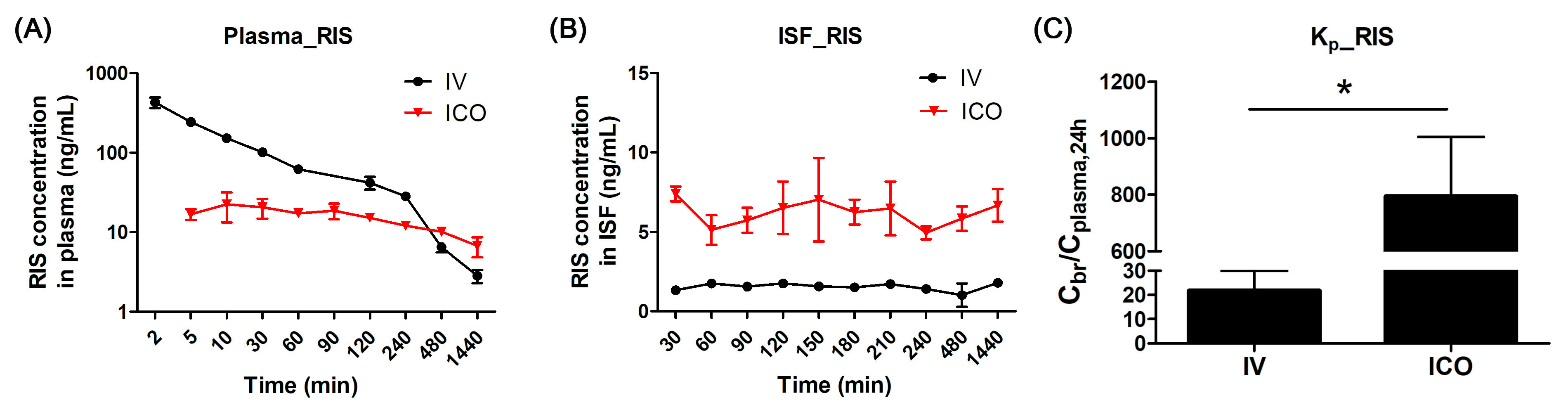


**Fig. S5.** The concentration-time profiles of risperidone (RIS) in the plasma (A) and ISF (B) following a single intravenous (IV) and intracalvariosseous (ICO) administration in mice. (C) Calculated ratio of brain/plasma concentration at 24 h (K_p_) after IV and ICO administration of RIS (mean±SEM, n=4).


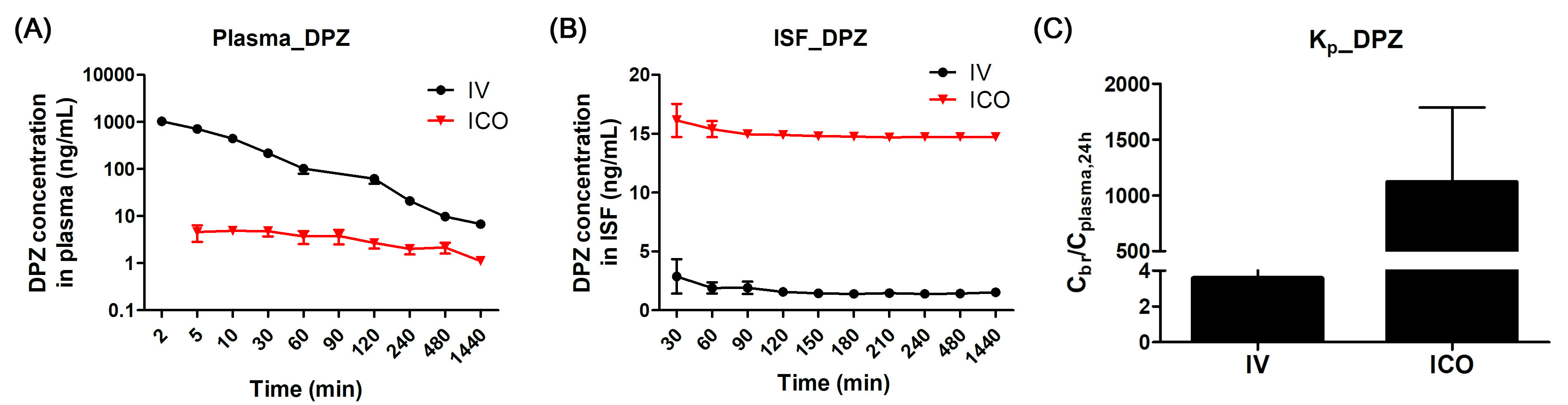


**Fig. S6.** The concentration-time profiles of donepezil (DPZ) in the plasma (A) and ISF (B) following a single intravenous (IV) and intracalvariosseous (ICO) administration in mice. (C) Calculated ratio of brain/plasma concentration at 24 h (K_p_) after IV and ICO administration of DPZ (mean±SEM, n=4).


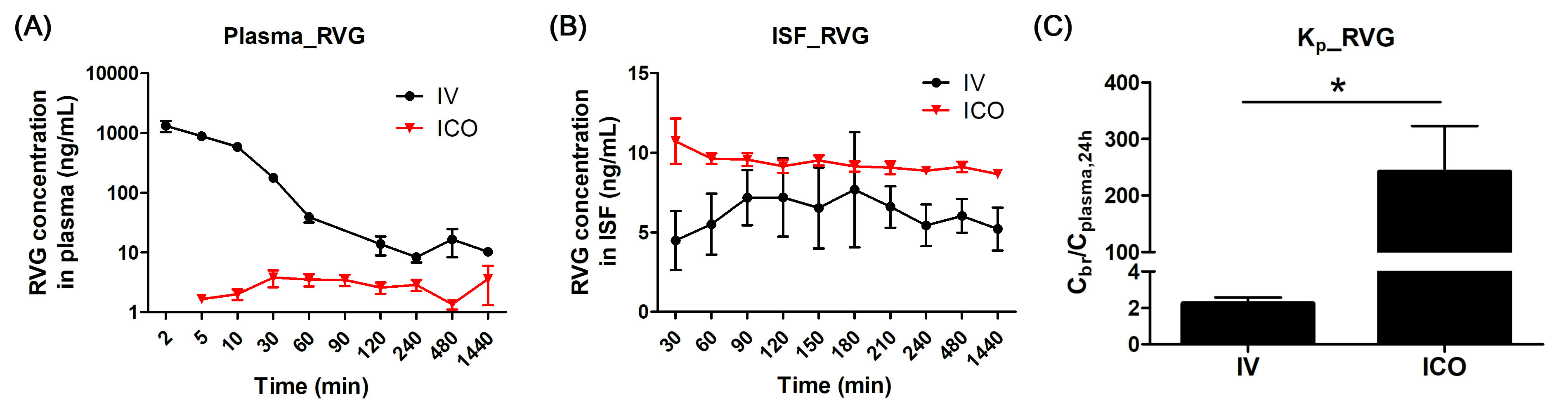


**Fig. S7.** The concentration-time profiles of rivastigmine (RVG) in the plasma (A) and ISF (B) following a single intravenous (IV) and intracalvariosseous (ICO) administration in mice. (C) Calculated ratio of brain/plasma concentration at 24 h (K_p_) after IV and ICO administration of RVG (mean±SEM, n=4).


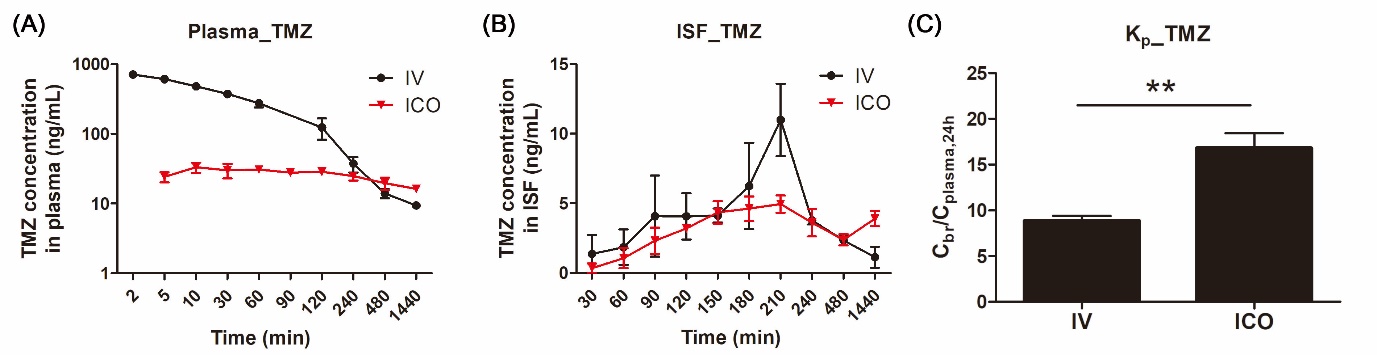


**Fig. S8.** The concentration-time profiles of temozolomide (TMZ) in the plasma (A) and ISF (B) following a single intravenous (IV) and intracalvariosseous (ICO) administration in mice. (C) Calculated ratio of brain/plasma concentration at 24 h (K_p_) after IV and ICO administration of TMZ (mean±SEM, n=4).


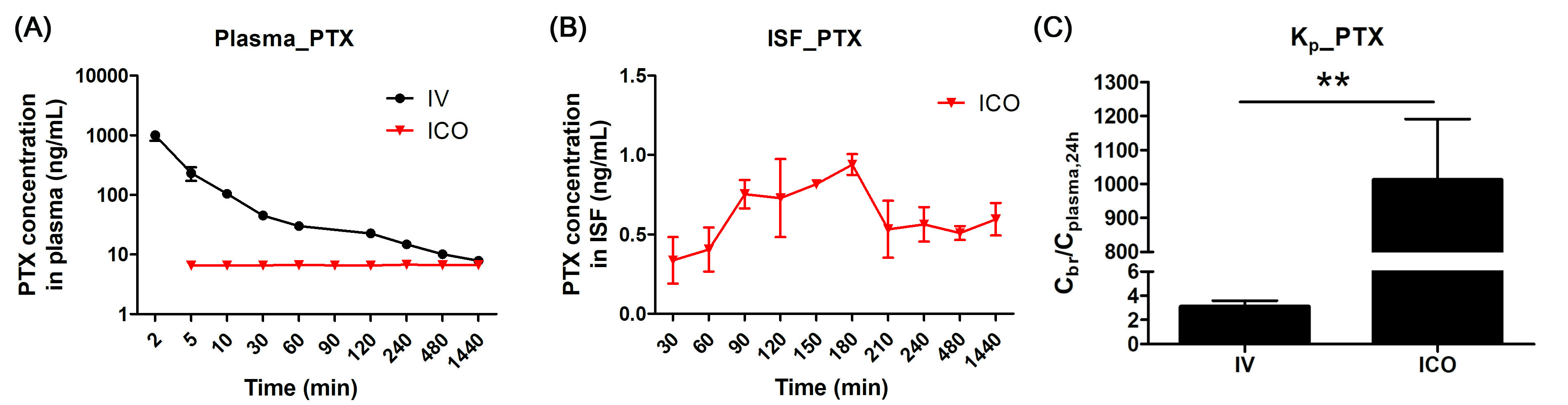


**Fig. S9.** The concentration-time profiles of paclitaxel (PTX) in the plasma (A) and ISF (B) following a single intravenous (IV) and intracalvariosseous (ICO) administration in mice. (C) Calculated ratio of brain/plasma concentration at 24 h (K_p_) after IV and ICO administration of PTX (mean±SEM, n=4).

**
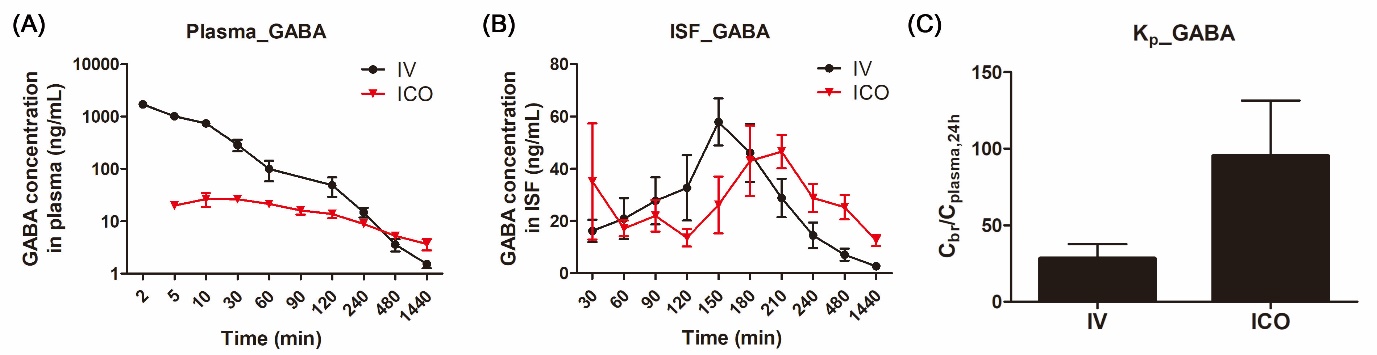
**

**Fig. S10.** The concentration-time profiles of γ-Aminobutyric Acid-d6 (GABA) in the plasma (A) and ISF (B) following a single intravenous (IV) and intracalvariosseous (ICO) administration in mice. (C) Calculated ratio of brain/plasma concentration at 24 h (K_p_) after IV and ICO administration of GABA (mean±SEM, n=4).


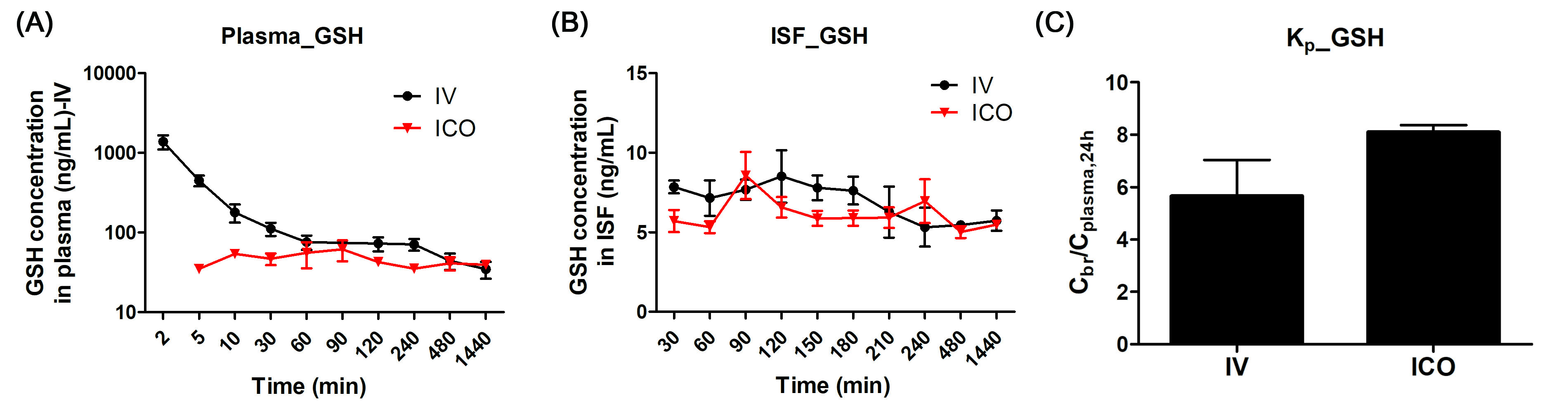


**Fig. S11.** The concentration-time profiles of glutathione (GSH) in the plasma (A) and ISF (B) following a single intravenous (IV) and intracalvariosseous (ICO) administration in mice. (C) Calculated ratio of brain/plasma concentration at 24 h (K_p_) after IV and ICO administration of GSH (mean±SEM, n=4).

**
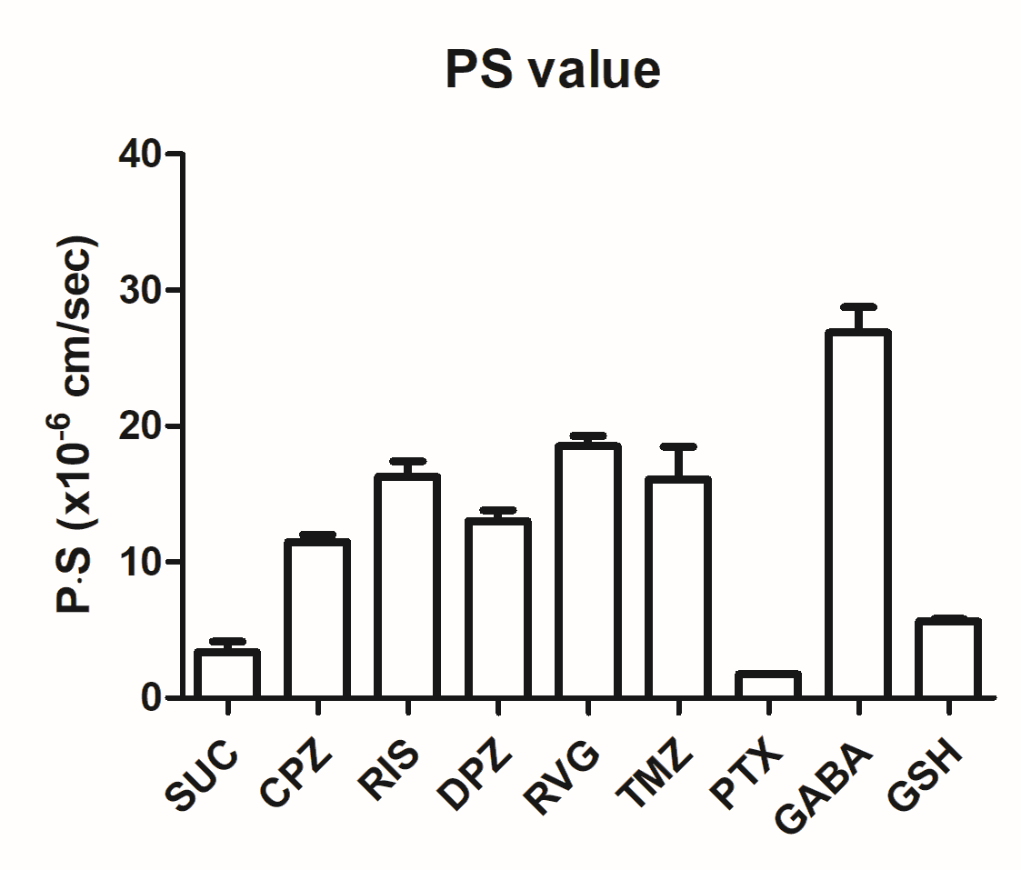
**

**Fig. S12**. The PS value of nine compounds across the *in vitro* BBB monolayers (mean±SEM, n=4).

**Table S2**. The calculated PS values, molecular weight and Log Pow of nine compounds (mean±SEM, n=4).

| Compound | PS | MW | Log Pow |
| --- | --- | --- | --- |
| SUC | 3.38 ± 0.77 | 342.300 | -3.70 [^3^](#_ENREF_3) |
| CPZ | 11.49 ± 0.51 | 318.860 | 5.41 [^4^](#_ENREF_4) |
| RIS | 16.27 ± 1.16 | 410.493 | 3.27 [^5^](#_ENREF_5) |
| DPZ | 13.02 ± 0.78 | 379.492 | 4.14 [^6^](#_ENREF_6) |
| RVG | 18.52 ± 0.75 | 250.342 | 2.30 [^7^](#_ENREF_7) |
| TMZ | 16.10 ± 2.41 | 194.151 | -1.15 [^7^](#_ENREF_7) |
| PTX | 1.74 ± 0.04 | 853.906 | 3.20 [^7^](#_ENREF_7) |
| GABA | 26.89 ± 1.89 | 103.120 | -3.17 [^3^](#_ENREF_3) |
| GSH | 5.65 ± 0.16 | 307.324 | -3.10 [^8^](#_ENREF_8) |

**Reference**

1. Zhou Q, Gallo JM. In vivo microdialysis for PK and PD studies of anticancer drugs. *The AAPS journal*. Oct 24 2005;7(3):E659-67. doi:10.1208/aapsj070366

2. Alqahtani F, Chowdhury EA, Bhattacharya R, Noorani B, Mehvar R, Bickel U. Brain Uptake of [(13)C] and [(14)C]Sucrose Quantified by Microdialysis and Whole Tissue Analysis in Mice. *Drug metabolism and disposition: the biological fate of chemicals*. Nov 2018;46(11):1514-1518. doi:10.1124/dmd.118.082909

3. Hansch C, Leo A, Hoekman D, eds. *Exploring QSAR: Hydrophobic, electronic, and steric constants*. American Chemical Society; 1995. Hansch C, Leo A, Hoekman DH, eds. *ACS Professional Reference Book*.

4. Frisk-Holmberg M, van der Kleijn E. The relationship between the lipophilic nature of tricyclic neuroleptics and antidepressants, and histamine release. *European journal of pharmacology*. May 1972;18(2):139-47. doi:10.1016/0014-2999(72)90235-x

5. Wang SJ, Lu HT, Wang YC, Huang HY, Yang CS. A rapid screening method to select microdialysis carriers for hydrophobic compounds. *PloS one*. 2021;16(9):e0256920. doi:10.1371/journal.pone.0256920

6. Yasir M, Sara UVS, Chauhan I, et al. Solid lipid nanoparticles for nose to brain delivery of donepezil: formulation, optimization by Box–Behnken design, in vitro and in vivo evaluation. *Artificial Cells, Nanomedicine, and Biotechnology*. 2018/11/17 2018;46(8):1838-1851. doi:10.1080/21691401.2017.1394872

7. Wishart DS, Feunang YD, Guo AC, et al. DrugBank 5.0: a major update to the DrugBank database for 2018. *Nucleic acids research*. Jan 4 2018;46(D1):D1074-d1082. doi:10.1093/nar/gkx1037

8. Liu M, Sharma M, Lu GL, et al. Preformulation studies of l-glutathione: physicochemical properties, degradation kinetics, and in vitro cytotoxicity investigations. *Drug development and industrial pharmacy*. May 2020;46(5):717-731. doi:10.1080/03639045.2020.1752708
